# Identification of Hub Genes and Their Correlation With Immune Infiltration Cells in Hepatocellular Carcinoma Based on GEO and TCGA Databases

**DOI:** 10.3389/fgene.2021.647353

**Published:** 2021-04-30

**Authors:** Rui Huang, Jinying Liu, Hui Li, Lierui Zheng, Haojun Jin, Yaqing Zhang, Wei Ma, Junhong Su, Min Wang, Kun Yang

**Affiliations:** ^1^College of Medicine, Northwest Minzu University, Lanzhou, China; ^2^Lanzhou Maternity and Child Health Care Hospital, Lanzhou, China; ^3^Medical Faculty, Kunming University of Science and Technology, Kunming, China; ^4^Lanzhou University Second Hospital, Lanzhou, China

**Keywords:** hepatocellular carcinoma, weighted gene co-expression network analysis, RobustRankAggreg, CIBERSORT, immune infiltration

## Abstract

Hepatocellular carcinoma (HCC) is a primary liver cancer with extremely high mortality in worldwide. HCC is hard to diagnose and has a poor prognosis due to the less understanding of the molecular pathological mechanisms and the regulation mechanism on immune cell infiltration during hepatocarcinogenesis. Herein, by performing multiple bioinformatics analysis methods, including the RobustRankAggreg (RRA) rank analysis, weighted gene co-expression network analysis (WGCNA), and a devolution algorithm (CIBERSORT), we first identified 14 hub genes (NDC80, DLGAP5, BUB1B, KIF20A, KIF2C, KIF11, NCAPG, NUSAP1, PBK, ASPM, FOXM1, TPX2, UBE2C, and PRC1) in HCC, whose expression levels were significantly up-regulated and negatively correlated with overall survival time. Moreover, we found that the expression of these hub genes was significantly positively correlated with immune infiltration cells, including regulatory T cells (Treg), T follicular helper (TFH) cells, macrophages M0, but negatively correlated with immune infiltration cells including monocytes. Among these hub genes, KIF2C and UBE2C showed the most significant correlation and were associated with immune cell infiltration in HCC, which was speculated as the potential prognostic biomarker for guiding immunotherapy.

## Introduction

Liver cancer is a highly malignant tumor with a worldwide mortality rate ranking among the top three in 2020 (Sung et al., 2021). Hepatocellular carcinoma (HCC) is a primary liver cancer that originates in the liver. The development of large-scale sequencing technology and subsequent big data mining methods has made a great contribution on the research on HCC genome changes. In addition to focusing on cancer cells, research on the infiltration of host immune cells has recently become the new concerns for cancer biology. Therefore, exploring the potential prognostic biomarkers and developing immunotherapy methods are crucial for improving the survival of cancer patients (Wei et al., 2020).

The Cancer Genome Atlas (TCGA) and Gene Expression Omnibus (GEO) databases are the two most commonly used tumor databases. It is necessary to repeatedly mine tumor databases given the continuous updates in bioinformatics technology. RobustRankAggreg (RRA) algorithm was extensively used to screen the differentially expressed genes (DEGs) in different dataset from multiple different sequencing platforms (Kolde et al., 2012). The R package for Weighted Correlation Network Analysis (WGCNA) was used to identify co-expressed gene modules with similar expression patterns, and to explore the relationship between gene networks and phenotypes of interest (Langfelder and Horvath, 2008). In the recent years, many reports were published for investigating the hub genes of HCC(Chen et al., 2017; Li et al., 2018; Zhou et al., 2019). Chen et al. (2017) identified 6 hub genes, *ABAT*, *AGXT*, *ALDH6A1*, *CYP4A11*, *DAO*, and *EHHADH*, that were associated with HCC metastasis risk. Li et al. (2018) identified the 5 hub genes, *GINS1*, *TOP2A*, *BUB1B*, *ARPC4*, and *ACADM* in HCC progression with high node degree. In this study, we comprehensively applied a variety of bioinformatics algorithms to screen out 171 HCC-related genes with significantly up- or down-regulated expression levels. Then, we have used protein-protein network ranked by 12 different algorithms of cytohubba to find 14 hub genes, whose expression levels were significantly up-regulated. Among them, the high expression level of 11 genes, NDC80, DLGAP5, BUB1B, KIF20A, KIF2C, KIF11, NCAPG, PBK, FOXM1, TPX2, and PRC1, were strongly correlated with shorter overall survival time. Furthermore, we carried out an immune cell infiltration analysis by CIBERSORT and found these hub genes was significantly positively correlated with immune infiltration cells, including regulatory T cells (Treg), T follicular helper (TFH) cells, macrophages M0, but negatively correlated with immune infiltration cells including monocytes. The results further illustrate that the expression of the hub genes is associated with a poor prognosis.

## Materials and Methods

The flow chart showing the overall research design and methods used for this study was shown in Supplementary Figure 1.

### GEO Data Collection and Preprocessing

The GEO database was searched by using the keywords “hepatocellular carcinoma” and “liver cell carcinoma.” After filtering according to the following criteria (1) all samples are from human beings, (2) all datasets include matched cancer tissue-normal tissue samples, and (3) the dataset contains at least 20 samples, 7 microarray data sets (GSE62322, GSE112790, GSE102079, GSE14323, GSE14520, GSE89377, and GSE64041) were selected and downloaded. The detail showed in Supplementary Table 1.

### TCGA Data Download and Preprocessing

Gene expression quantification data and corresponding clinical information for HCC were downloaded from The Cancer Genome Atlas Liver Hepatocellular Carcinoma (TCGA-LIHC) data collection. The 424 HTSeq-counts files comprised 371 tumor samples and 50 normal samples. Clinical information was extracted and included follow-up time and clinical status. The TCGA expression matrix was obtained by data fusion and ID transformation of raw TCGA counts data. Next, the RPKM (Reads Per Kilobase per Million mapped reads) values were calculated for the WGCNA.

We applied the “limma” package (Ritchie et al., 2015) of R software to perform normalization and base-2 logarithm conversion for the matrix data for each GEO and TCGA dataset. differentially expressed genes for each GEO and TCGA matrix were obtained by transforming expression values, and genes were sorted according to the log_2_FoldChange (logFC) value. Next, rank analysis was performed using the R package “RRA.” The criterion for screening DEGs is that the *P* < 0.05 and | logFC | > 1.

### Identification of Gene Expression Modules

GEO data from the same platform was merged as follows. The GPL570 array platform included three datasets GSE62322, GSE112790, and GSE102079. GSE14323 and GSE14520 were merged into the GPL571 platform. Using the “sva” package the batch effect and other unwanted variations were removed to avoid generating less reliable results (Leek et al., 2012). Next, we selected gene expression matrixes from GPL570, GPL571, GSE89377, GSE64041, and RPKM values of TCGA data and then identified gene expression modules using the WGCNA package in R. Setting an appropriate soft-thresholding power to ensure scale-free networks, *R*^2^ = 0.9 was selected. The adjacency matrix used to construct the Topological Overlap Matrix (TOM) using TOM similarity values and module eigengenes (MEs) were clustered using the dissimilarity measure (1-TOM).

### Gene Ontology (GO) and Kyoto Encyclopedia of Genes and Genomes (KEGG) Pathways Enrichment Analysis

DEGs for GO and KEGG pathway enrichment analysis consist of two datasets. The first included 101 overlapping genes from 3 components the including integrated DEGs (RRA_diff), MEs with the strongest positive tumor correlation of GEO (GEO_positive ME) and TCGA (TCGA_positive ME), the other dataset included 70 overlapping genes integrating DEGs (RRA_diff), MEs with the strongest negative tumor correlation of GEO (GEO_ negative ME), and TCGA (TCGA_ negative ME). The merged 171 genes were retained for GO and KEGG pathway enrichment analysis using R packages. *P* < 0.05 was considered statistically significant.

### Functional Protein Association PPI (Protein-Protein Interaction) Network Analysis

The overlapping genes were analyzed to identify potential interactions using the online STRING database (Szklarczyk et al., 2017). PPIs with the highest confidence scores ≥ 0.9 were reserved and the results were imported to Cytoscape (Shannon et al., 2003) for further complex network analysis. Moreover, to predict and explore important hub genes in the PPI network, we performed module analysis utilizing the cytohubba application with default parameters in Cytoscape. Overlapping of 12 topological algorithms were carried out using the “UpSetR” package. Finally, 14 overlapping genes were obtained.

### Survival Analysis

The clinical information of patients with HCC was extracted and included follow-up time and clinical status. After removing patients with no information on overall survival (OS) data, candidate genes strongly correlated with survival were identified using the “survival” and “survminer” packages.

### Correlation Between Survival-Related Candidate Genes and Immune Cells

To study the correlation between survival-related candidate genes and immune cells, we used a web server called TIMER2.0^1^ (Taiwen et al., 2020). We detected the correlation in the expression of 14 hub genes in HCC with the levels of infiltrating immune cells, respectively, using the CIBERSORT as the deconvolution algorithm. The 22 immune cells included T cells, B cells, macrophages, dendritic cells (DCs), NK cell, monocytes, mast cells, eosinophils, and neutrophils. The correlation between them was shown using a heat map.

### Infiltrating Immune Cells Between Tumor and Normal Samples

We compared the infiltrating immune cells of tumor and normal samples. The original gene expression data from TCGA were normalized as described previously (Chen et al., 2018). The normalized data was analyzed using the R package “CIBERSORT.” CIBERSORT, a deconvolution algorithm based on principles of linear support vector regression, was published in Nature Methods in 2015. It calculated the cell composition of unknown mixture based on their gene expression profiles according to known reference set LM22 (Leukocyte signature matrix). This reference dataset defines the gene expression characterizing a set of signature genes of 22 immune cell subtypes (Newman et al., 2015). The permutations (perm) of the deconvolution algorithm were set at 100. The results were filtered using a *p* < 0.05.

All data were processed using R language (version 4.0.2) and all statistical methods in this study were performed using corresponding R package. When the *p* < 0.05, results were considered statistically significant. Researchers who want the R code can contact the corresponding author (yangkun@lzu.edu.cn).

## Results

### Identification of Integrated GEO DEGs and TCGA DEGs

**FIGURE 1:**
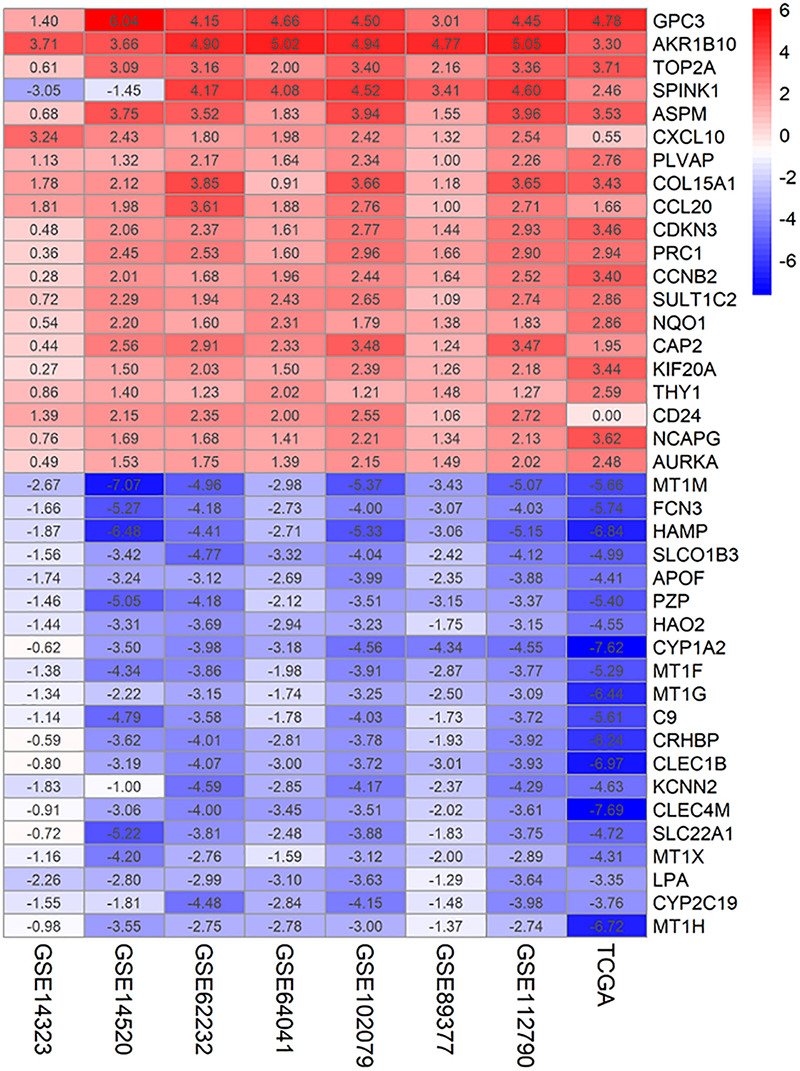
Identification of DEGs of 7 GEO datasets and TCGA dataset using RRA. The heatmap of the top 20 up- and down-regulated DEGs in the integrated GEO datasets analysis. The rows represent the genes and the columns represent the GEO dataset. The number in the rectangle represents the log_2_FoldChange value. Red represents up-regulated genes, blue represents down-regulated genes.

Seven microarray data sets [GSE62322 (Schulze et al., 2015), GSE112790 (Shimada et al., 2019), GSE102079 (Chiyonobu et al., 2018), GSE14323 (Mas et al., 2009), GSE14520 (Roessler et al., 2010), GSE89377, and GSE64041 (Makowska et al., 2016)] and the TCGA-LIHC dataset were downloaded and processed. Data sets from different platforms cannot be merged directly using “limma” packages for differentially expressed genes analysis. Therefore, we ranked the 8 data sets from different platforms using the RRA algorithm. Finally, 199 significantly up-regulated genes and 363 significantly down-regulated genes were identified by the RRA method. Top 20 up-regulated and down-regulated genes are shown in Figure 1. The | logFC| > 1 and adjusted *p* < 0.05 were considered statistically significant for the RRA DEGs.

### Identification of Key Gene Expression Modules

**FIGURE 2:**
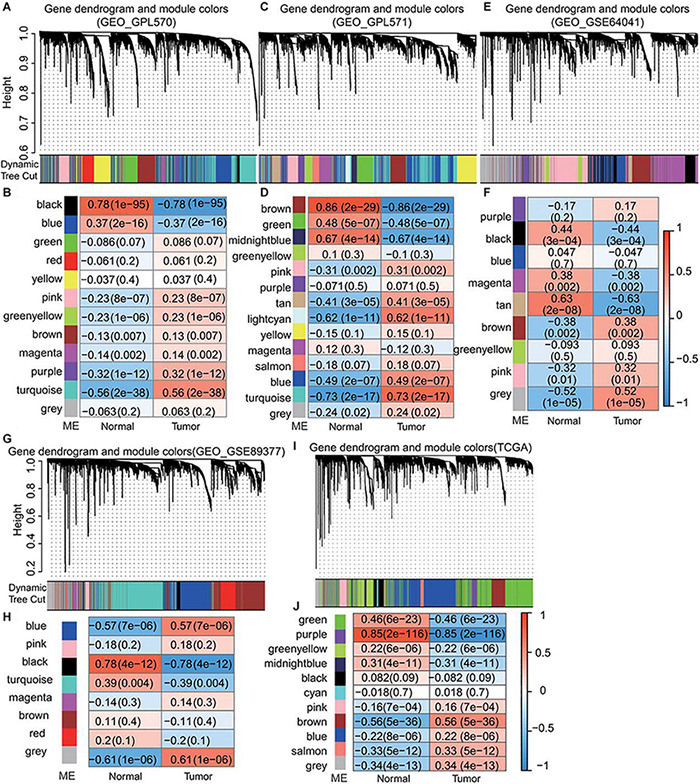
Identification of module eigengenes (MEs) associated with HCC using GEO and TCGA datasets. **(A**,**C**,**E**,**G**,**I)** Dendrogram of DEGs clustered based on a dissimilarity measure (1–TOM). **(B**,**D**,**F**,**H**,**J)** Module-trait relationships. Each row corresponds to a color module and each column correlates to a clinical trait (normal and cancer). The numbers in each cell represent the corresponding correlation and *P*-value.

GEO data from the same platform were merged. GSE62322, GSE112790, and GSE102079 were merged into the GPL570 platform and GSE14323 and GSE14520 were merged into the GPL571 platform. Then, we selected gene matrixes from GPL570, GPL571, GSE89377, GSE64041, and RPKM values of TCGA data and constructed a co-expression network with the WGCNA package in R. By gathering similarly expressed genes in tumor and normal tissue, mRNAs with similar expression profiles were aggregated into the different module by applying a dynamic tree cut algorithm. The module-trait relationships are shown in Figure 2. Next, 650 module eigengenes having the strongest positive tumor correlation from the GEO were merged to form GEO_positive ME including MEturquoise of GPL570, MEturquoise of GPL571, MEgrey of GSE64041, and MEgrey of GSE89377. 222 module eigengenes with the strongest tumor negative correlation were merged to form the GEO_negative ME and included MEblack of GPL570, MEbrown of GPL571, MEtan of GSE64041, and MEblack of GSE89377. The results showed in Supplementary Table 2.

### GO and KEGG Pathway Enrichment Analysis of DEGs

**FIGURE 3:**
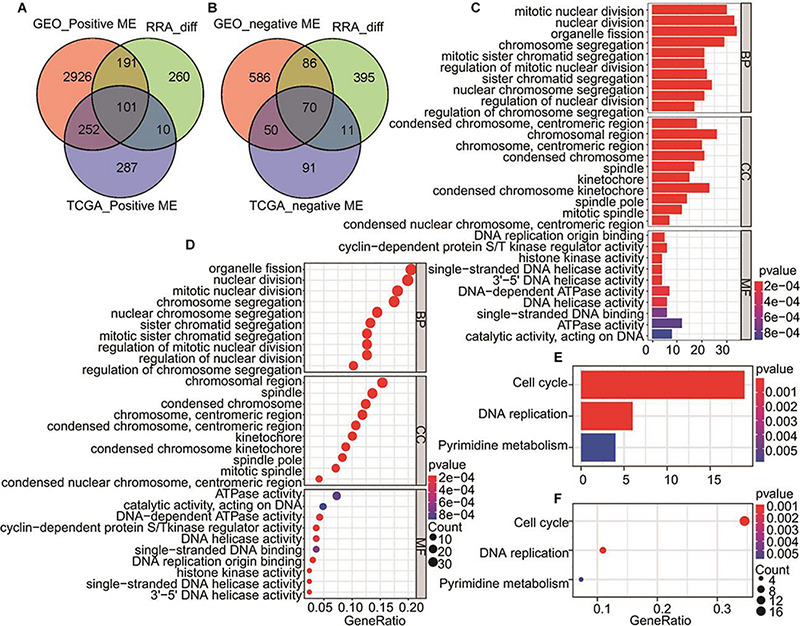
Venn diagrams for overlapping DEGs and MEs significantly related to HCC and overlapped genes for GO and KEGG pathways analysis. **(A)** Tumor positive related genes. GEO_ positive MEs includes MEturquoise of GPL570, MEturquoise of GPL571, MEgrey of GSE64041, and MEgrey of GSE89377. **(B)** Tumor negative related genes. GEO_negative MEs includes MEblack of GPL570, MEbrown of GPL571, MEtan of GSE64041, and MEblack of GSE89377. **(C)** The bar plot of all overlapping genes by GO biological process. **(D)** The bubble plot showing all overlapping genes by GO biological process **(E)** KEGG pathways of all overlapped genes are shown in the bar plot. **(F)** KEGG pathways of all overlapped genes showed in the bubble plot.

171 gene including 101 genes from GEO_positive ME, TCGA_positive ME, and RRA DEGs (Figure 3A) and 70 genes from GEO_negative ME, TCGA_negative ME, and RRA DEGs (Figure 3B) were merged for GO and KEGG pathway enrichment analysis. The results showed the genes were involved in the biological process (BP), cell component (CC), molecular function (MF). We found the biological processes in which these genes are mainly involved include nuclear division, chromosome segregation and organelle fission, of these, the expression products were mainly components of the chromosome, spindle and kinetochore (Figures 3C,D). KEGG analysis showed that these genes are involved in signal pathways including the cell cycle, DNA replication and pyrimidine metabolism (Figures 3E,F).

### Identification of Hub Genes

**FIGURE 4:**
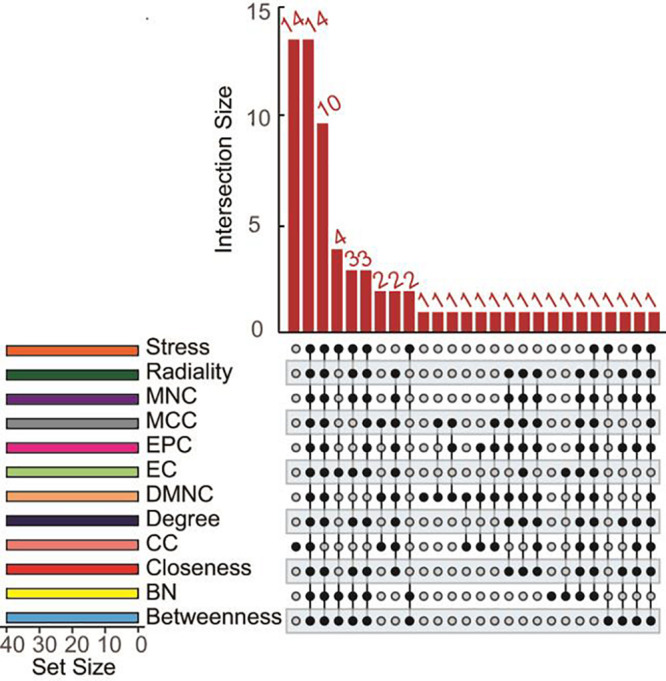
UpSet diagrams of 12 topological algorithms determined by functional protein association PPI network analysis. The overlapping results of several topological algorithms including Degree, EPC, MNC, DMNC, MCC, and network centralities based on the shortest paths such as BN, EC, Closeness, Radiality, Betweenness, Stress and CC are shown.

In total, 171 overlapped genes were analyzed to characterize the potential protein-protein interactions using the online STRING database. PPIs with a highest confidence score ≥ 0.9 were selected and then imported to cytoscape for further complex network analysis. In addition, to predict and explore the important hub genes in the PPI network, we used cytoHubba with default parameters in cytoscape. Firstly, we found many genes are ranked same if just ranked by one algorithm (Supplementary Table 3). So, we thought that the parameters calculated using multiple algorithms maybe reflect the status of the node in the entire network from different aspects. Then, we got top 40 genes from protein-protein network ranked by 12 different algorithms of cytohubba including Degree, Density of Maximum Neighborhood Component (DMNC), Edge Percolated Component (EPC), Maximal Clique Centrality (MCC), Maximum Neighborhood Component (MNC), and centralities based on shortest paths, such as Bottleneck (BN), Closeness, EcCentricity (EC), Radiality, Betweenness, Stress, and Clustering Coefficient (CC) were intersected. Finally, 14 hub genes (degree value > 20), NDC80, DLGAP5, BUB1B, KIF20A, KIF2C, KIF11, NCAPG, NUSAP1, PBK, ASPM, FOXM1, TPX2, UBE2C, and PRC1 were obtained for further exploration (Figure 4). Details of these 14 hub genes was showed in Table 1.

**TABLE 1:** Details of hub genes.

**Name**	**RRA_logFC**	**Functions**
NDC80	1.86	Core element of kinetochores, function in Kinetochore-Microtubule Attachment (Ciferri et al., 2008)
DLGAP5	1.74	Also known as DLGAP7/HURP (hepatoma up-regulated protein), a kinetochore protein that can be regulated by phosphorylation of AURKA, stabilizes microtubules (Surhone et al., 2011; Tagal et al., 2017).
BUB1B	1.79	Spindle assembly checkpoint protein, directly bind to CDC20 to inhibit anaphase-promoting complex activity (Chen, 2002)
KIF20A	1.82	Kinesin family member 20A, involved in cytokinesis (Neef et al., 2003)
KIF2C	1.32	Kinesin family member 2C, Mediates the depolymerization at plus end of microtubules thereby promotes the separation of chromosome during mitosis (Bakhoum et al., 2009)
KIF11	1.16	kinesin family member 11 (also known as Eg5). Function in centrosome migration and spindle bipolarity during cell mitosis (Rapley et al., 2008)
NCAPG	1.86	Is necessary for chromosome condensation (Kimura et al., 2001)
NUSAP1	2.10	Nucleolar spindle-associated protein (Raemaekers et al., 2003)
PBK	1.90	MAPKK-like protein kinase, may be involved in the activation of lymphoid cells, maintain testicular functions (Abe et al., 2000)
ASPM	2.85	Is essential for spindle regulation (Kouprina et al., 2005)
FOXM1	1.35	Transcription factor, Cyclin regulatory protein (Laoukili et al., 2005)
TPX2	1.31	Microtubule nucleation factor, spindle assembly, activation of AURKA (Bird and Hyman, 2008)
UBE2C	1.44	Ubiquitin conjugating enzyme E2, regulate destruction of cyclins in mitotic (Townsley et al., 1997)
PRC1	2.17	Participate in cytokinesis (Jiang et al., 1998)

### The Relationship Between Hub Gene and Overall Survival Time (OS)

**FIGURE 5:**
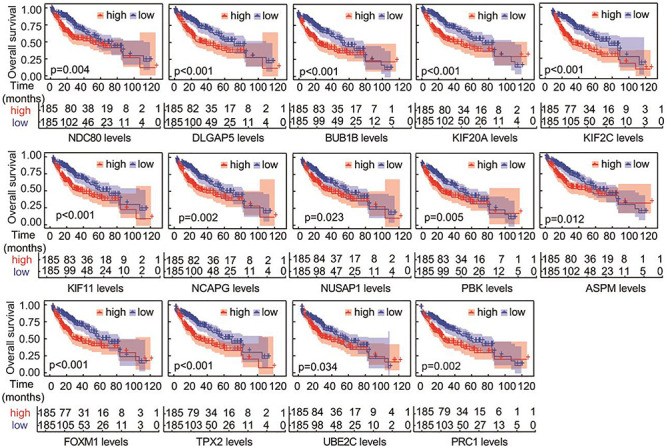
Overall survival analyses of the overlapping hub genes. Analysis was performed using the survival and survminer packages in R. *P*-values were used to indicate significant differences.

The OS of the 14 hub genes was analyzed by survival and survminer package. The results demonstrated that there was an extremely significant correlation between the expression levels of DLGAP5, BUB1B, KIF20A, KIF2C, KIF11, FOXM1, and TPX2 and survival time (*p* < 0.001), a significant correlation existed between the expression of NDC80, NCAPG, PBK, PRC1, and survival time (*p* < 0.01), and a weak correlation between the expression of NUSAP1, ASPM, UBE2C, and survival (*p* < 0.05) (Figure 5).

### Correlation Between Survival-Related Candidate Genes and Immune Cells

**FIGURE 6:**
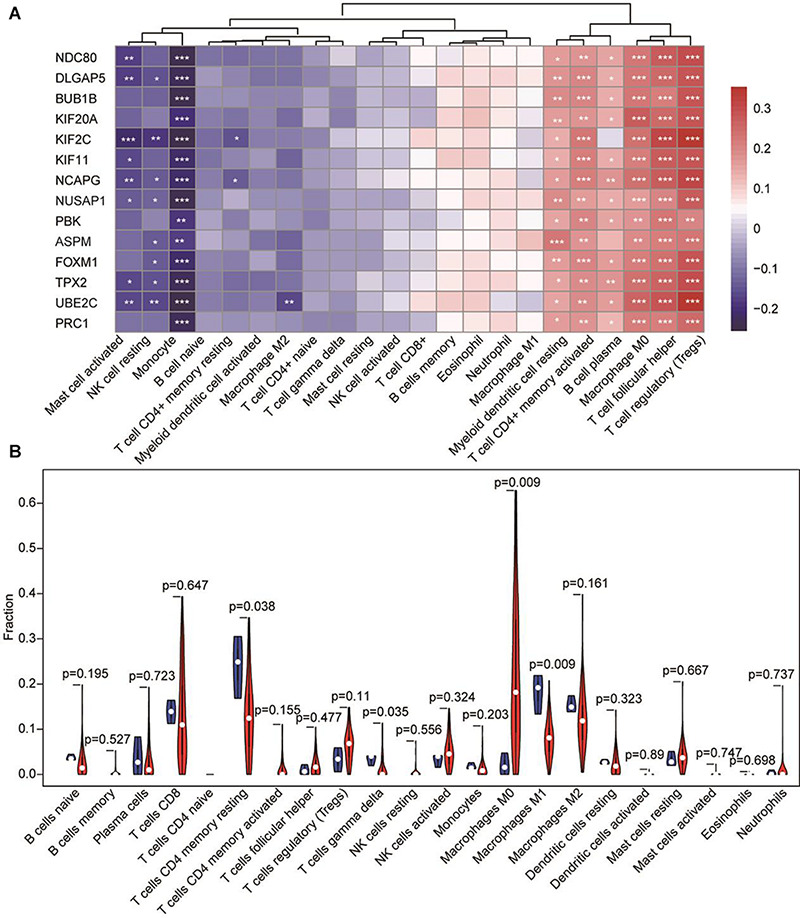
The correlation between hub genes-immune cells and the fraction of infiltrated immune cells between normal tissue and tumor tissue in HCC. **(A)** The expression of candidate hub genes having significant positive correlations with immune infiltration cells are shown in red. The expression of candidate hub genes having significant negative correlations with immune infiltration cells are shown in blue. *p < 0.05, **p < 0.01, *** p < 0.001. **(B)** Fraction of infiltrated immune cells in HCC. Red represents tumor tissue and blue shows normal tissue.

Next, we explored whether the mRNA expression level of the candidate hub genes was associated with infiltrating immune cells in HCC. Thus, we compared the expression of the 14 candidate hub genes in HCC and the infiltrating levels of immune cells using the TIMER database. The results showed that the expression level of candidate hub genes was significantly positively correlated with the infiltrating levels of immune cells including Treg cells, TFH cells, macrophages (M0), T cell CD4 + memory activated, myeloid dendritic cell resting and B cell plasma, and significantly negative correlations with immune infiltrating cells including monocytes, mast cells activated and NK cell resting. In addition, very weak negative correlations were existed between T cell CD4 + memory resting and the hub genes (Figure 6A). More interestingly, KIF2C and UBE2C showed the most significant positive correlation with Treg and a negative correlation with immune infiltration of monocytes (Supplementary Table 4). Meanwhile, we compared the fraction of infiltrating immune cells with tumor and normal tissues and found the content of macrophages M0 in tumor tissues significantly higher than that in normal tissue, while the fraction of macrophages M1 showed opposite result to macrophages M0 (Figure 6B).

## Discussion

Tumorigenesis is a very complex process which includes the changes in expression of various genes and changes in the microenvironment. It is vital for tumor therapy to identify novel biomarkers and targets and to explore the diversity and complexity of the tumor immune microenvironment. At present, this complicated process can be opportunely explored because of the development of high-throughput sequencing technology and subsequent data analysis techniques. Herein, we have screened the hub genes in HCC and revealed a strong correlation between hub genes expression level and the tumor microenvironment.

We identified 14 hub genes (NDC80, DLGAP5, BUB1B, KIF20A, KIF2C, KIF11, NCAPG, NUSAP1, PBK, ASPM, FOXM1, TPX2, UBE2C, and PRC1) that were significantly associated with overall survival by RRA rank analysis and WGCNA based on TCGA and GEO databases. Previous studies showed that 14 hub gene are involved in cell cycle regulation, which is according with our results from GO and KEGG pathway enriched analysis. It has previously been reported that some of these also regulate the P53 signaling pathway (Mirgayazova et al., 2019). The hub genes have been also found to be associated with various cancers (Burum-Auensen et al., 2010; Wierstra, 2013; Luo et al., 2014; Singh et al., 2014; Schneider et al., 2017; Gao and Wang, 2019). In this study, we found the expression of the hub genes was up-regulated in cancer samples compared with normal tissues. Some hub genes, such as DLGAP5 and TPX2, whose activation was also regulated by the other up-regulated genes (Bird and Hyman, 2008; Tagal et al., 2017). Interestingly, the high expression level of NDC80, DLGAP5, BUB1B, KIF20A, KIF2C, KIF11, NCAPG, PBK, FOXM1, TPX2, and PRC1 corresponds to a short overall survival time.

The most interesting finding in this study is that there is a correlation between the expression level of hub genes and immune infiltrating cells. It is well-known that infiltrating immune cells, are a component of the tumor microenvironment and play an important role in tumor growth, invasion, and metastasis. The tumor microenvironment has diverse capacities to induce both adverse and beneficial consequences for tumorigenesis (Quail and Joyce, 2013). Our study showed that there was a significantly positive correlation between the expression of hub genes and infiltrating immune cells including Treg, TFH, and macrophages (M0), but the most significant negative correlation was with monocytes.

It has previously been reported that Tregs and TFH cells exert opposite roles in tumorigenesis. Tregs suppress antitumor immunity. In contrast, TFH cell contribute to antitumor immunity (Zhang and Zhang, 2020). Tregs are present in a variety of tumors and are physiologically involved in the maintenance of immunological self-tolerance through immunosuppressive effects, thereby allowing tumor cells to escape the body’s immune killing (Nishikawa and Sakaguchi, 2010; Finotello and Trajanoski, 2017). TFH cells are an independent subset of CD4 + T effector cells having an essential role in assisting B cell proliferation and antibody production (Ochando and Braza, 2017). We observed that the expression level of KIF2C and UBE2C are strongly positively corelatedcorrelated to the content of Tregs and TFH. It has previously been reported that T cells from patients with metastatic melanoma could recognize mutated kinesin family member 2C (KIF2C) antigen (Lu et al., 2014). But there was no significant difference between the Treg or TFH infiltrating immune cell density when comparing tumor and normal cells.

However, the density of M0 macrophages in tumor tissues was significantly increased compared with normal tissues, in contrast, the density of M1 macrophages in tumor tissues was significantly decreased. Macrophages infiltrating in tumors act as a “double-edged sword” in tumorigenesis and development. M1 type macrophages can kill tumor cells, while M2 type macrophages promote tumor growth. Our findings indicating whether it is the up-regulation in expression level of the hub gene or the decrease macrophage M1 levels, it will induce adverse consequences for tumorigenesis. Meanwhile, we observed the expression level of hub genes negatively correlated with monocyte levels. Macrophages are partially differentiated from monocytes in peripheral blood in response to a wide spectrum of growth factors and chemokines produced by stromal and tumor cells (Noy and Pollard, 2014). Monocytes in the tumor microenvironment may induce beneficial consequences for tumorigenesis. Therefore, we thought that the hub genes induced adverse consequences for tumorigenesis and can be used the potential prognostic biomarker.

## Conclusion

In conclusion, our study, based on several bioinformatics algorithms, revealed hub genes and their correlation with immune infiltration cells in HCC and our comprehensive analysis identified associations between 22 immune cells subpopulations. We found 11 genes, *NDC80, DLGAP5, BUB1B, KIF20A, KIF2C, KIF11, NCAPG, PBK, FOXM1, TPX2*, and *PRC1*, were strongly associated with poor prognosis. Nonetheless, our findings still require further experimental validation in the future.

## Data Availability Statement

The datasets presented in this study can be found in online repositories. The names of the repository/repositories and accession number(s) can be found in the article/Supplementary Material.

## Author Contributions

RH and KY conceived and designed the study and wrote the manuscript. RH, JL, HL, LZ, YZ, HJ, WM, JS, and MW analyzed the data. RH, JL, HL, LZ, HJ, YZ, WM, JS, MW, and KY revised the manuscript. All authors have read and approved the final version of the manuscript.

## Conflict of Interest

The authors declare that the research was conducted in the absence of any commercial or financial relationships that could be construed as a potential conflict of interest.

## Footnotes

1 https://cistrome.shinyapps.io/timer/

## Supplementary Material

Supplementary Figure 1 Flowchart detailing the overall study design and samples at each stage of analysis.

Supplementary Table 1 Information for the seven GEO datasets included in the current study.

Supplementary Table 2 MEs related to HCC.

Supplementary Table 3 Top 40 genes from protein-protein network ranked by 12 different algorithms of cytohubba including Degree, DMNC, EPC, MCC, MNC, Bottleneck, Closeness, EcCentricity, Radiality, Betweenness, Stress and Clustering Coefficient.

Supplementary Table 4 The correlation between survival-related candidate genes and immune cells. Spearman’s Rho and *p*-values were calculated using the online web resource TIMER 2.0.

## Abbreviations

HCC: Hepatocellular carcinoma
RRA: RobustRankAggreg
WGCNA: weighted gene co-expression network analysis
Treg: regulatory T cell; TFH T follicular helper cells
LM22: Leukocyte signature matrix
GEO: Gene Expression Omnibus
DEGs: Differentially expressed genes
TCGA: The Cancer Genome Atlas
RPKM: Reads Per Kilobase per Million mapped reads
TOM: Topological Overlap Matrix
MEs: module eigengenes
EPC: Edge Percolated Component
MNC: Maximum Neighborhood Component
DMNC: Density of Maximum Neighborhood Component
MCC: Maximal Clique Centrality
BN: Bottleneck
CC: Clustering Coefficient
OS: overall survival
BP: biological process
CC: cell component
MF: molecular function.
