# Supplementary figures and images for: Identification of Hub Genes and Their Correlation With Immune Infiltration Cells in Hepatocellular Carcinoma Based on GEO and TCGA Databases
Source: Front Genet. 2021 Apr 30;12:647353. doi: 10.3389/fgene.2021.647353 (PMC8120231; doi:10.3389/fgene.2021.647353)

**Figure S1**

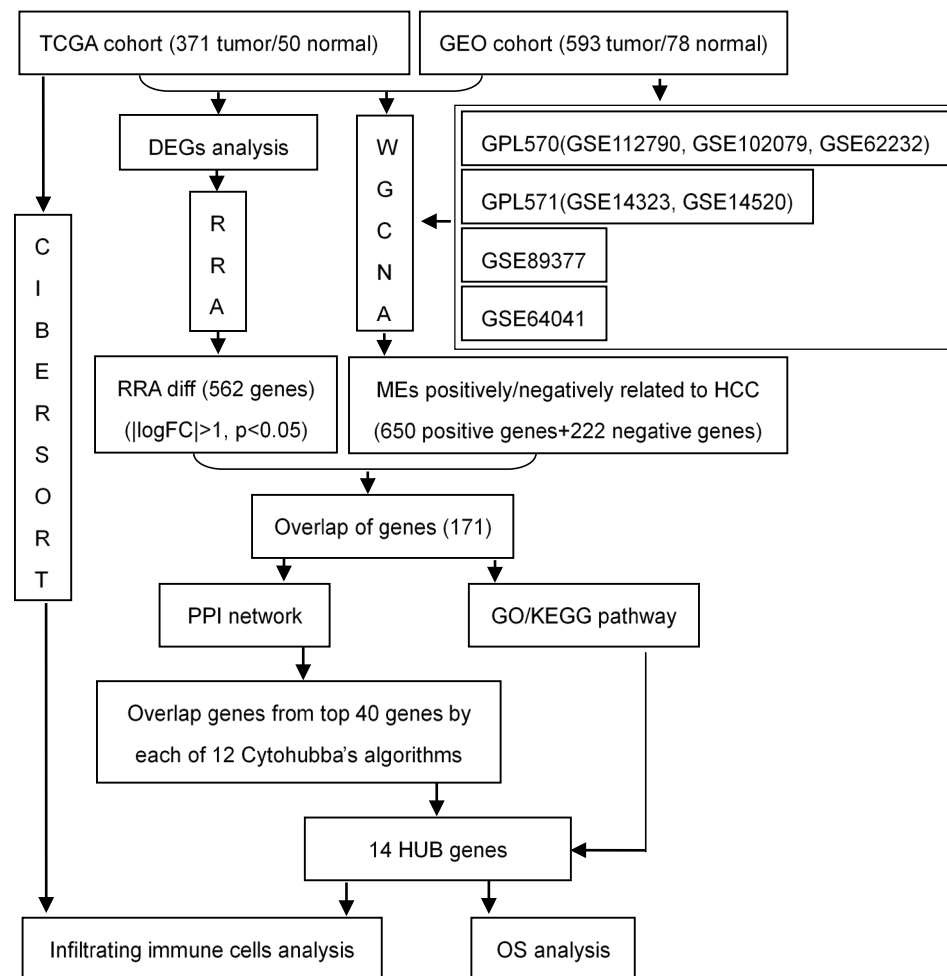

Supplement: Supplementary Figure 1 — Flowchart detailing the overall study design and samples at each stage of analysis. [file Image_1.pdf]
