# Supplementary material for: Identification of Hub Genes and Their Correlation With Immune Infiltration Cells in Hepatocellular Carcinoma Based on GEO and TCGA Databases
Source: Front Genet. 2021 Apr 30;12:647353. doi: 10.3389/fgene.2021.647353 (PMC8120231; doi:10.3389/fgene.2021.647353)
Supplement: Supplementary Table 1 — Information for the seven GEO datasets included in the current study. [file Table_1.DOCX]

**Table S1 Information for the seven GEO datasets included in the current study**

| Reference | Dataset | Platform | Number of samples (Tumor/Control) |
| --- | --- | --- | --- |
| ([Schulze et al., 2015](#_ENREF_5)) | GSE62322 | GPL570 [HG-U133_Plus_2] Affymetrix Human Genome U133 Plus 2.0 Array | 91(81/10) |
| ([Shimada et al., 2019](#_ENREF_6)) | GSE112790 | GPL570 [HG-U133_Plus_2] Affymetrix Human Genome U133 Plus 2.0 Array | 198 (183/15) |
| ([Chiyonobu et al., 2018](#_ENREF_1)) | GSE102079 | GPL570 [HG-U133_Plus_2] Affymetrix Human Genome U133 Plus 2.0 Array | 166 (152/14) |
| ([Mas et al., 2009](#_ENREF_3)) | GSE14323 | GPL571 [HG-U133A_2] Affymetrix Human Genome U133A 2.0 Array | 74 (55/19) |
| ([Roessler et al., 2010](#_ENREF_4)) | GSE14520 | GPL571 [HG-U133A_2] Affymetrix Human Genome U133A 2.0 Array | 24 (22/2) |
| - | GSE89377 | GPL6947 Illumina HumanHT-12 V3.0 expression beadchip | 53 (40/13) |
| ([Makowska et al., 2016](#_ENREF_2)) | GSE64041 | GPL6244 [HuGene-1_0-st] Affymetrix Human Gene 1.0 ST Array | 65 (60/5) |
