# Supplementary material for: Identification of Hub Genes and Their Correlation With Immune Infiltration Cells in Hepatocellular Carcinoma Based on GEO and TCGA Databases
Source: Front Genet. 2021 Apr 30;12:647353. doi: 10.3389/fgene.2021.647353 (PMC8120231; doi:10.3389/fgene.2021.647353)
Supplement: Supplementary Table 2 — MEs related to HCC. [file Table_2.DOCX]

**Table S2 MEs related to HCC**

|  |  |  | ME positively related to HCC | ME negatively related to HCC |
| --- | --- | --- | --- | --- |
| GEO | GPL_570 | GSE112790 | turquoise | black |
|  |  | GSE102079 |  |  |
|  |  | GSE62232 |  |  |
|  | GPL571 | GSE14323 | turquoise | brown |
|  |  | GSE14520 |  |  |
|  |  | GSE64041 | grey | tan |
|  |  | GSE89377 | grey | black |
| TCGA | - | - | brown | purple |
